# Contrastive-Active Transfer Learning-Based Real-Time Adaptive Assessment Method for Power System Transient Stability

**DOI:** 10.3390/s24155052

**Published:** 2024-08-04

**Authors:** Jinman Zhao, Xiaoqing Han, Chengmin Wang, Jing Yang, Gengwu Zhang

**Affiliations:** 1 College of Electrical and Power Engineering, Taiyuan University of Technology, 79 Yingze West Street, Taiyuan 030024, China; 2 Key Laboratory of Control of Power Transmission and Conversion (SJTU), Ministry of Education, 800 Dongchuan Road, Minhang District, Shanghai 200240, China; 3 College of Automotive Engineering, Shanxi Vocational University of Engineering Science and Technology, 369 Wenhua Street, Yuci District, Jinzhong 030619, China

**Keywords:** transient stabilization assessment, contrastive learning, active learning, transfer learning

## Abstract

The transient stability assessment based on machine learning faces challenges such as sample data imbalance and poor generalization. To address these problems, this paper proposes an intelligent enhancement method for real-time adaptive assessment of transient stability. In the offline phase, a convolutional neural network (CNN) is used as the base classifier. A model training method based on contrastive learning is introduced, aiming to increase the spatial distance between positive and negative samples in the mapping space. This approach effectively improves the accuracy of the model in recognizing unbalanced samples. In the online phase, when real data with different distribution characteristics from the offline data are encountered, an active transfer strategy is employed to update the model. New system samples are obtained through instance transfer from the original system, and an active sampling strategy considering uncertainty is designed to continuously select high-value samples from the new system for labeling. The model parameters are then updated by fine-tuning. This approach drastically reduces the cost of updating while improving the model’s adaptability. Experiments on the IEEE39-node system verify the effectiveness of the proposed method.

## 1. Introduction

Transient stability assessment is an important research topic in power system security and stability analysis [1,2]. With the large-scale grid connection of renewable energy sources, the flexibility and complexity of power systems are increasing, and the shortcomings of traditional power system transient stability assessment methods, such as large computational volume and low computational efficiency, are becoming more and more prominent, and the search for more efficient and accurate transient stability assessment methods has become the focus of current research [3,4].

As new-generation artificial intelligence technologies like deep learning and deep reinforcement learning continue to advance, data-driven approaches have emerged as innovative methodologies for power system research [5,6,7]. Artificial intelligence technology establishes the mapping relationship between power system operation characteristics and transient stability results from the data perspective to achieve fast and accurate end-to-end assessment [8,9,10,11]. Reference [12] selects sample information features through mutual information and Pearson coefficients, and reference [13] uses a random forest algorithm to assess the security of the system by filtering features through mutual information and Pearson coefficients. Reference [14] proposed a dynamic security assessment (DSA) framework based on a conditional Bayesian deep autoencoder. Reference [15] proposed a dynamic security assessment method based on integrated modeling. DSA is oriented towards transient stability preventive control, which refers to the ability of the power system operating state to withstand certain disturbances.

All of the above methods achieved high assessment accuracy. However, considering the low likelihood of accidents in real power systems, when artificial intelligence methods such as deep learning are used, they face the problem of an imbalance in the number of stabilized and unstable samples, which in turn affects the performance of the classifiers. To solve this problem, some researchers have introduced the generative adversarial network (GAN) [16,17,18] into transient stability assessment with sample imbalance. Reference [19] proposed an improved GAN algorithm to generate unstable data and reduce the impact of unbalanced datasets on model training. Reference [20] uses conditional generative adversarial networks to expand the unstable samples and construct sample-balanced datasets. Considering that unsupervised learning GAN has the problem of generating uncontrollable samples, this paper introduces contrastive learning into transient stability assessment and proposes a transient stability assessment algorithm based on contrastive learning.

Contrastive learning is an emerging machine learning algorithm that trains models by maximizing the similarity of positive sample pairs while minimizing the similarity of negative sample pairs, which can effectively improve the model’s generalization ability on complex datasets. Since 2019, it has been widely used in several fields, including computer vision, natural language processing, and graph learning, and is recommended in several fields [21,22,23]. Traditional supervised learning uses cross-entropy as the loss function, and although it performs well in most computations, it is susceptible to noise, less robust, and not applicable to high-risk application scenarios, such as class imbalance. Contrastive learning helps to enhance the expression ability of the model in the feature space by emphasizing the differences and similarities between the learning samples, which is especially suitable for the sample proportion imbalance scenario. In the field of power systems, the application of contrastive learning is still in the preliminary stage. Reference [24] establishes a neural network-based line fault localization framework that applies contrastive learning to the classification of raw signals. Reference [25] proposes a graph attention contrastive learning framework that utilizes instance-level contrast loss to characterize the similarity of models and enhance model evaluation.

In addition, existing data-driven transient stabilization studies rely on a complete sample library in the offline phase. When the grid operation mode or topology changes significantly, the model built based on the original topology is not applicable, and a new sample database is needed to train the model. Transfer learning is a machine learning method that utilizes existing knowledge to improve the performance of models in new scenarios [26,27,28]. Transfer learning has proven effective in addressing model performance degradation caused by significant variations in operating conditions within the context of DSA. Reference [29] uses transfer learning methods to improve model performance under new operating conditions, and reference [30] uses fine-tuning methods in transfer learning to improve model generalizability by fixing the shallow parameters of the model while keeping the structure of the model unchanged and using the knowledge learned by the model in the source domain to assist in updating, thus reducing the updating cost. However, when the distribution difference between the source and target domains is large, the frozen source domain model parameters are not favorable for learning new feature representations in the target domain. To address the above issues, this paper combines active learning to further enhance the transferability of the model by selecting the most representative target domain samples for fine-tuning through active learning, a strategy that can better help the model learn the feature space distribution of the target domain with limited annotation resources.

This paper presents a real-time adaptive evaluation method for DSA that employs contrastive-active transfer learning. The offline phase uses contrastive learning to enhance the model’s generalization ability to unbalanced data, which in turn improves the DSA model’s performance. In the online assessment phase, when the operating conditions of the power system change, the active transfer strategy updates the model, thereby minimizing the number of samples needed for updating while maintaining transfer performance and effectively addressing the issue of small sample updates in the power system.

## 2. Real-Time Adaptive Assessment of Power System Transient Stability

**Figure 1:**
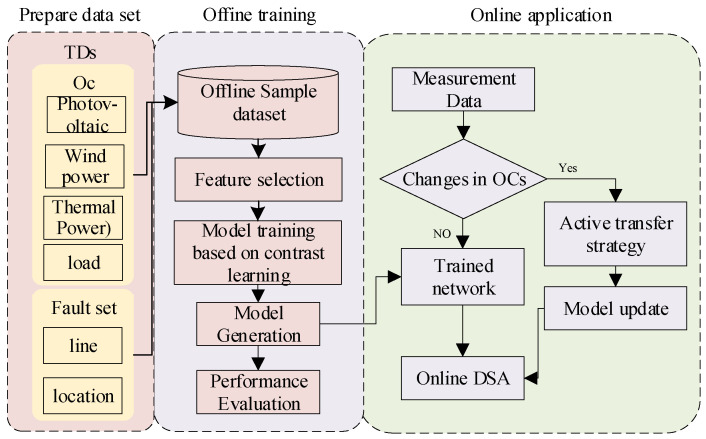
Overall framework diagram.

The application of data-driven methods in the DSA of power systems mainly consists of two phases: offline training and online application. In the offline phase, the model learns and builds a prediction model for simulation data. Although the training process may be time-consuming, the time overhead in this phase does not directly affect the efficiency of the online application. In the online application, the sampling interval of the measurement device is 10 ms, and the system extracts features from the measurement data and inputs them into the model every 10 ms. The average prediction time of the model is at the millisecond level, which can satisfy the system’s demand for real-time response. Aiming at the imbalance of the ratio of stable and unstable samples and the problem of model generalizability in transient stability assessment. In this paper, we propose a real-time adaptive assessment method for DSA based on contrast-active transfer learning. In the offline phase, the model’s expressive ability in the face of unbalanced data is enhanced by contrastive learning. In the online phase, an active transfer strategy is used to enhance the model’s performance under new operating conditions. The overall framework is illustrated in Figure 1.

### 2.1. Sample Set Construction

According to the DSA literature, DSA requires the grid to have the ability to withstand specific events, and in practice, the tolerable event is set to be a fault with a duration of 0.1 s. In order to ensure the comprehensiveness of the data, this paper considers the output fluctuations of wind power and photovoltaic units, generates samples by changing the proportion of the corresponding thermal power units, and performs dynamic “N-1” calibration on all fault lines and batch simulation.

In this paper, the transient stability index (TSI) is selected as the transient stability assessment index.

(1) $$\eta =\frac{\pi -|\Delta \delta_{\max}|}{\pi +|\Delta \delta_{\max}|}$$

Where $\Delta \delta_{\max}$ is the maximum power angle difference between thermal power units, when η>0 denotes transient stability, η<0 denotes transient instability. Assuming that there are n lines for dynamic “N-1” verification, the sample label can be expressed as:

(2) $$\mathrm{label}=\{\begin{array}{l} 1,\;\mathrm{if}\;\cap_{i=1}^{n}(\eta_{i}>0)\\ 0,\;\mathrm{otherwise}\; \end{array}$$

If the current mode of operation satisfies the dynamic “N-1” fault checks for all lines, the label is 1. Conversely, if one of the dynamic “N-1” checks fails, the label is 0.

### 2.2. Feature Extraction

Considering that the DSA in the pre-fault scenario pays more attention to the distribution of the system’s tidal current, this paper adopts the steady-state features as the input features of the model. Considering that feature selection in machine learning has an important impact on the performance of machine learning models, this paper constructs several feature subsets as alternative feature sets, from which the optimal feature subsets will be selected as model input features, as shown in Table 1.

The filtered optimal subset will be used as feature input to the model, and in this paper, convolutional neural network (CNN), a classical deep learning model, is selected as the base classifier for transient stability assessment. Through convolution operation and pooling operation, CNN can significantly improve the feature extraction ability of the model on the input data. The convolutional operation enables the model to locally perceive the input data and capture local features, while the pooling operation helps to reduce the data dimensionality and improve the computational efficiency of the model. In addition, the horizon-sharing mechanism of CNN allows the model to share weight parameters at different locations, which reduces the number of parameters of the network and improves the generalization performance of the model.

## 3. Contrastive Learning

### 3.1. Contrastive Learning

Contrastive learning is a machine learning model based on sample similarity, and its core idea lies in learning feature representations by comparing similarities and differences between data samples. Contrastive learning is mainly applied to self-supervised learning in early research, which utilizes data structure to guide feature learning by self-generating and comparing data, after which researchers extended it to the field of supervised learning with labels, which assists the contrastive learning process with the help of labeling information to improve the model performance further.

In traditional supervised learning, the model relies on explicit labeling information to guide model training, whereas contrastive learning does not require explicit labeling; it learns feature representations from the structure of the data itself. The research paradigm of contrastive learning consists of defining agent tasks and objective function guidance. Firstly, begin with the original data $x_{i}$ and perform data augmentation tasks to create enhanced data $x_{i}^{\prime }$. Subsequently, construct positive and negative sample pairs from these augmented datasets. Typically, sample pairs derived from the same original sample via different augmentation techniques are categorized as positive pairs, while pairs generated from different original samples are considered negative pairs. Secondly, compute the model loss using the objective function to guide the model’s learning process. The aim is to maximize the similarity within positive pairs and minimize the similarity within negative pairs.

Let N denote the number of samples in a batch, the number of samples after expansion is 2N, except for the current positive samples, the other 2(N-1) sample pairs are negative sample pairs. The distance between samples is measured by cosine similarity, so that u and v denote the representation vectors of two samples in the feature space respectively, and the distance between pairs of samples based on cosine similarity can be expressed as:

(3) $$\mathrm{sim}(u,v)=\frac{u^{T}v}{\left\|u\|\;\|v\right\|}$$

To avoid the computational complexity associated with a large number of negative sample pairs, info noise contrastive estimation (NCE) is used in self-supervised learning to compare the loss function and maximize the probabilistic optimization model of correct sample pairs through log-likelihood estimation.

(4) $$ℒ=\frac{1}{N}\sum_{i\in T}-\log\frac{\exp[\mathrm{sim}(z_{i}\cdot z_{j(i)})/\tau ]}{\sum_{a\in A_{i}}\exp[\mathrm{sim}(z_{i}\cdot z_{a})/\tau ]}$$

where the numerator is the distance of positive sample pairs and the denominator is the distance of negative sample pairs. $z_{i}$ is the feature representation of the sample, $z_{j(i)}$ denotes the positive sample feature representation after data enhancement, $z_{a}$ is the negative sample feature representation, and τ is the temperature coefficient.

### 3.2. Supervised Contrastive LEARNING

The cross-entropy (CE) function is often used as the loss function in traditional supervised learning, so that *c* denotes the number of sample categories, $y_{i}$ denotes the sample category, ${\hat{y}}_{i}$ denotes the model prediction output, the loss function can be expressed as:

(5) $${ℒ}_{CE}=-\frac{1}{N}\sum_{i=1}^{N}\sum_{c=1}^{C}y_{i}\cdot \log{\hat{y}}_{i}$$

**Figure 2:**
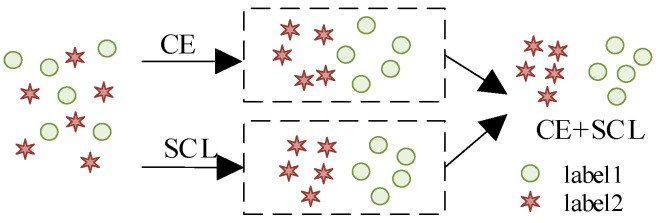
Comparison between CE loss and SCL loss.

The CE loss function mainly focuses on minimizing the distance of intraclass samples and ignores maximizing the distance of interclass samples. Supervised contrastive learning (SCL) adds the maximization of interclass distance on this basis, which is able to maximize the similarity of similar labeled samples and minimize the similarity of labeled samples of different classes at the same time, so as to further optimize the model performance, and take into account the two perspectives of intraclass and interclass [31,32]. As shown in Figure 2.

In the classification task, SCL can directly define positive and negative sample pairs based on labels, where samples of the same class are regarded as positive samples and samples of different classes are regarded as negative samples. Let λ be the weight coefficient that balances the cross-entropy loss and the supervised contrastive learning loss, and the total loss function is expressed as:

(6) $$ℒ=(1-\lambda ){ℒ}_{CE}+\lambda {ℒ}_{SCL}$$

Let $P_{i}$ denote the indexed set of all positive samples that belong to the same category as sample *i*, where ${ℒ}_{SCL}$ is the supervised comparison loss, defined as:

(7) $${ℒ}_{SCL}=\frac{1}{N}\sum_{i\in ℐ}\frac{1}{\left|P_{i}\right|}\sum_{p\in P_{i}}-\log\frac{\exp\left(z_{i}\cdot z_{p}/\tau \right)}{\sum_{a\in A_{i}}\exp\left(z_{i}\cdot z_{a}/\tau \right)}$$

To ensure the accuracy of data enhancement, this paper uses 1% Gaussian noise for data enhancement, and this noise magnitude is between 10^−3^ and 10^−2^, which is much smaller than the value of input features. Considering that the sample labels on the transient stability boundary of the power system are susceptible to perturbations, this paper selects to enhance only the samples near the center of the category. In this paper, the Euclidean distance is taken to describe the similarity of the samples in the feature space, and the augmented samples can be expressed as:

(8) $$x_{i}^{\prime }=1\left\|x_{i}-x_{i,c}\right\|<\delta \cdot \left(x_{i}+\epsilon \right)$$

where $x_{i,c}$ denotes the sample category center, ϵ is Gaussian noise, **1** is the schematic function, and δ is the threshold between the sample and the category center.

Assuming the number of enhanced samples is *M*, the total loss function after data enhancement can be expanded as:

(9) $$\begin{array}{ll} {ℒ}_{SCL} & =\;(1-\lambda )\sum_{i=1}^{N}\sum_{c=1}^{C}y_{i}\cdot \log{\hat{y}}_{i}\\ & +\;\lambda \frac{1}{N+M}(\sum_{\in ℐ}^{N+M}\frac{1}{\left|P_{i}\right|}\sum_{p\in P_{i}}-\log\frac{\exp\left(z_{i}\cdot z_{p}/\tau \right)}{\sum_{a\in A_{i}}\exp\left(z_{i}\cdot z_{a}/\tau \right)}) \end{array}$$

## 4. Adaptive Updating Strategies Based on Active Transfer Learning

### 4.1. Transfer Learning

**Figure 3:**
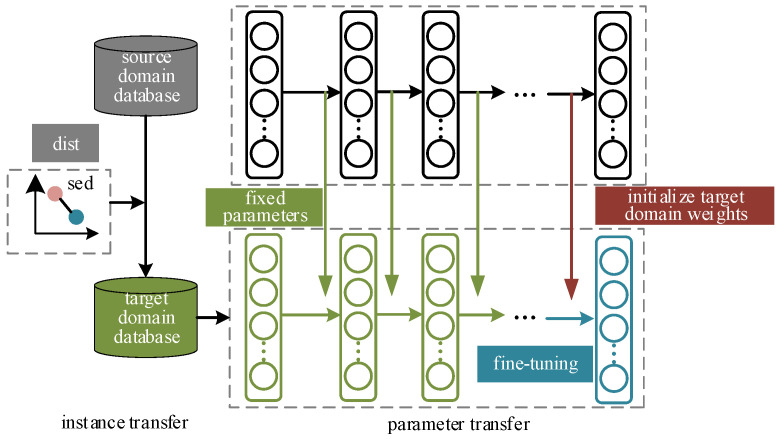
The process for instance-based and parameter-based transfer learning technique.

In the DSA problem, when offline-trained prediction models are applied to an online power system, problems such as insufficient model adaptation and reduced accuracy will occur once the online data distribution characteristics change. Transfer learning can improve the model adaptability by adjusting the model or feature space to fit the new data distribution. According to the different pathways, transfer learning is divided into methods, such as instance transfer, feature transfer, representation transfer, and parameter transfer. Instance transfer and parameter transfer are two commonly used algorithms, instance transfer can directly utilize the source domain samples to learn, and parameter transfer can be trained on the basis of existing parameters, which is conducive to rapid improvement of model performance. In this paper, we adopt a combination of instance transfer and parameter transfer to improve the performance of the model in new scenes. As shown in Figure 3.

The goal of instance transfer is to expand the sample pool of the target domain to provide more data support for subsequent model training. The samples from the source domain that are most similar to the target domain and can be migrated are selected according to the minimum distance principle. Denote the source domain samples as $x^{S}$, the target domain samples as $x^{T}$, and the selected samples can be denoted as:

(10) $$\{\begin{array}{l} \mathrm{dist}(x^{S},x^{T})=\mathrm{sed}(x^{S},x^{T})\\ {\overset{⌢}{x}}^{S}=\{x^{S}|\mathrm{dist}(x^{S},x^{T})<\theta \} \end{array}$$

where ${\overset{⌢}{x}}^{S}$ denotes the selected samples, and *θ* is the distance threshold. The selected samples will be used as the first step of model updating.

In parameter transfer, fine-tuning is a common strategy that utilizes the features learned by the source domain model in the task of interest and adapts to the data distribution in the target domain by making a small amount of adjustments on the target domain. The feasibility of fine-tuning in deep neural networks is demonstrated in the literature [33], where experiments discuss the characteristics and transferability of features extracted at each layer of the neural network, i.e., the feature extractors at the bottom layer usually capture generic features, while the classifiers at the top layer are more task-specific Relevance. This study provides a theoretical basis for the effectiveness of fine-tuning strategies.

### 4.2. Active Transfer Learning

Active learning improves the performance of the model by choosing to select the most informative samples to reduce the need for labeled samples. In active learning, the algorithm selects the most informative samples from unlabeled samples through some query strategy, sends them to experts for labeling to obtain their true labels, and then trains the model based on the labeled samples, updating the model through continuous iterative training until the target model reaches the preset performance.

Query strategy is the core element in active learning, which directly affects the quality of the selected samples. Uncertainty sampling is a commonly used sampling strategy, which can directly utilize the information entropy to quantify the uncertainty of the samples without additional distance metric calculation. The uncertainty metric prioritizes the samples with the largest information entropy, i.e., near the fuzzy classification boundary, which helps the model to distinguish the classification boundary between different samples.

(11) $$x^{*}=\arg\max_{x\in U}-\sum_{i}p\left(y_{i}|x\right)\log p\left(y_{i}|x\right)$$

where $x^{*}$ is the uncertain sample and $p\left(y_{i}|x\right)$ denotes the probability that the sample *x* belongs to the label $y_{i}$. In the active learning process, the samples will be selected from the unlabeled dataset *U* each time to obtain the labels through a time-domain simulation, which will be used for model updating later.

### 4.3. Adaptive Update Strategy Based on Active Transfer Learning

**Figure 4:**
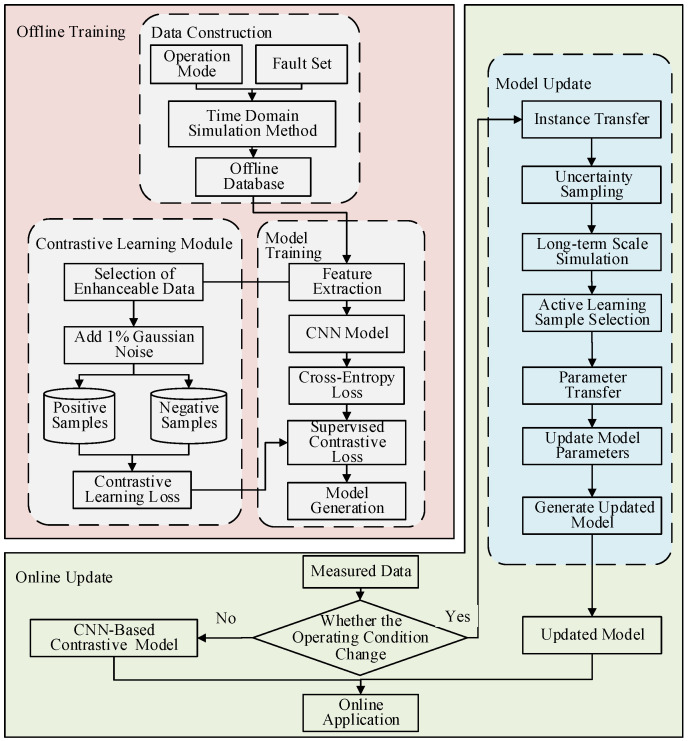
Adaptive assessment process for transient stability of power system.

The process of power system transient stability adaptive assessment utilizing contrastive learning-assisted training is depicted in Figure 4. This process primarily consists of two components: offline training and online evaluation.

In the offline process, the model constructs the simulation database by batch setting the operation mode and fault conditions. These extracted features are then utilized to train the model. To further improve model generalization, this paper introduces a new contrastive learning assistance module. Firstly, data enhancement is performed on the feature vectors that satisfy the conditions to obtain more positive and negative samples. Then, the network parameters of the feature extraction module are trained with the objective of supervised contrastive learning loss function. The mapping spatial distance between positive and negative samples in the feature space is pulled apart by maximizing the similarity measure between pairs of similar samples and minimizing the similarity measure between pairs of dissimilar samples. The loss value is calculated by supervised comparison loss, and the model parameters are calculated based on gradient descent to obtain the CNN-based transient stability assessment model.

In the online process, the measured data from the synchronized phasor measurement unit (PMU) is used as the input data, and the prediction model needs to be updated on time when the topology and operation mode of the system change. First, instance transfer is utilized to obtain high-quality data, and the samples in the source domain that are most similar to the samples in the target domain and can be migrated are selected as the starting point for expanding the sample pool in the target domain. Then, shallow weights are frozen to fix the first few layers of the network structure and parameters of the source domain CNN model. Finally, the samples are selected using active learning, and the uncertainty sampling strategy is used to select high-value data, and the sample labels are obtained through time-domain simulation, and the parameters of the target-domain CNN model are fine-tuned based on the selected data, fixing the shallow network parameters and updating the network parameters of the last layer only to obtain better evaluation performance.

### 4.4. Evaluation Indicators

In the actual operation of the power system, the number of actual destabilization samples is much less than the actual stabilization samples. In order to accurately assess the classifier, a confusion matrix is introduced to define the relevant indexes of its accuracy, as shown in Table 2. Where TP and TN represent the count of samples accurately predicted as stable and unstable by the model, respectively, while FP and FN indicate the count of unstable samples missed by it.

The evaluation metrics used in this paper contain *A*_cc_, *T*_TP_, *T*_TN_, and *F*_1_ 4 metrics, which comprehensively reflect the model evaluation performance.

(12) $$A_{\mathrm{cc}}=\frac{TP+TN}{TP+FP+FN+TN}\times 100\%$$

(13) $$T_{\mathrm{TP}}=\frac{TP}{TP+FN}\times 100\%$$

(14) $$T_{\mathrm{TN}}=\frac{TN}{TN+FP}\times 100\%$$

(15) $$F_{1}=\frac{2TP}{2TP+FP+FN}\times 100\%$$

## 5. Results

### 5.1. IEEE39 Node Experiment

**Figure 5:**
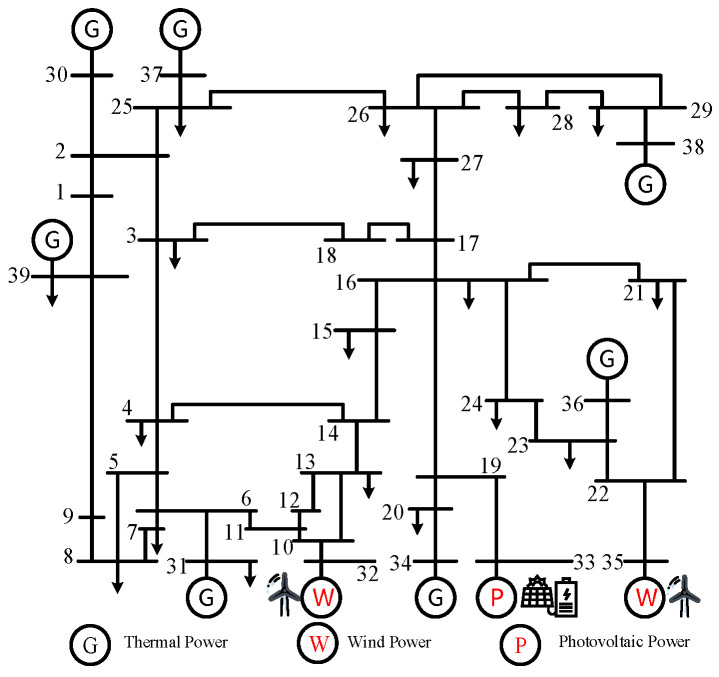
IEEE39 node structure.

In this paper, the IEEE39 node system is used as an example. The generator model is described as a second-order classical model, and the load model is described as a constant impedance. The Power System Analysis Software Package (PSASP) is used for the simulation, and the sampling step is set to 0.01 s. Considering the impact of renewable energy volatility on the system, the generator BUS-33 is replaced with a photovoltaic power plant. Generators BUS-32 and BUS-36 are replaced with wind turbines, as shown in Figure 5. The standard parameters given by the system component parameter arithmetic example, on the basis of which the thermal unit output is set to vary in 20% steps from 80% to 120%, and the wind turbine and PV unit output from 0% to 80% in 20% steps. Accordingly, the proportion of load is adjusted to ensure power balance, and the voltage is maintained at 0.95~1.05 (standardized value), and a permanent three-phase short-circuit fault of 0.1 s is set up for 34 transmission lines without transformers, with the fault locations of 2%, 50%, and 98%, respectively, and the simulation length is 5 s in total.

#### 5.1.1. Feature Subset Screening

In order to select the optimal feature subset, this paper sets to compare the performance of the transient stability assessment model under different input features, choosing CNN as the base classifier, with a convolutional kernel of 5, a maximal pooling layer connected with a step of 1 after each convolutional layer, and three fully connected layers to constitute. As can be seen from Table 3, the accuracy of the model is only 94.33 when feature subset 1 is used as the input feature, the accuracy of the model reaches 97.8 and all the indicators perform well when feature subset 2 and feature subset 3 are used as the input features, and the model accuracy reaches 97.8 and all the indicators perform well. When feature subset 4 is used as input features, the state identification accuracy of the model decreases to 96.87. This is due to the fact that feature subset 44 may contain a large number of low-information features, which affects the model’s state identification accuracy. a large number of low-information features, which affects the model’s performance.

**Figure 6:**
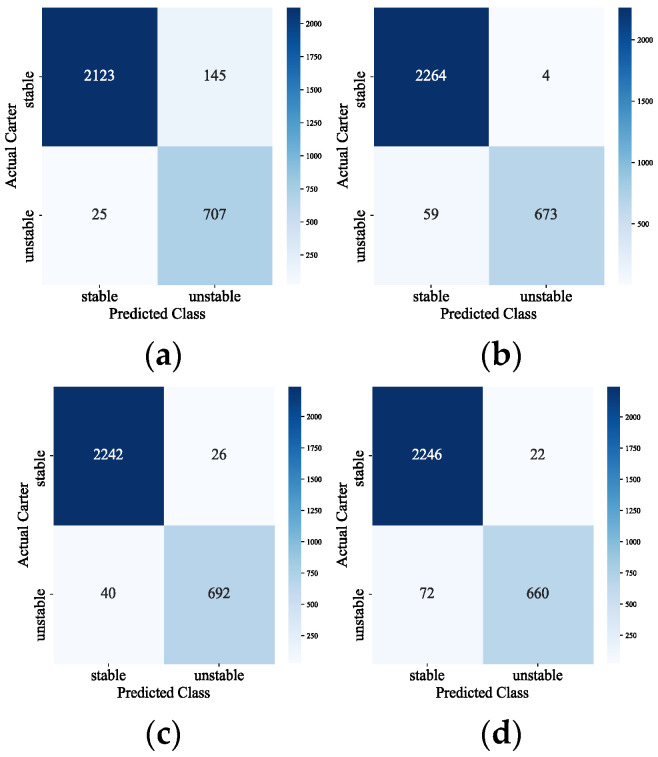
Schematic diagram of the confusion matrix. (**a**) Feature subset 1; (**b**) feature subset 2; (**c**) Feature subset 3; (**d**) feature subset 4.

To compare the confusion matrix of the model with different input features s, refer to Figure 6. The horizontal axis indicates the sample categories predicted by the model, and the vertical axis indicates the real categories of the samples. The larger the value of the main diagonal of the confusion matrix, and the smaller the value of the non-main diagonal region, the better the model prediction effect is. As can be seen in Figure 6, only four stable samples of the effect of feature subset 2 are misjudged as unstable samples, and the number of combined misjudged samples is 63, which is the least among the four feature subsets. In summary, feature subset two is chosen as the input features of the model.

#### 5.1.2. Model Performance Evaluation

Compare the performance of multiple typical data-driven algorithms with CNN models, as illustrated in Table 4. The experimental results indicate that, compared with the deep model, RF, a traditional machine learning method, performs poorly and is not applicable in transient stabilization assessment, and deep learning models such as MLP, LSTM, and CNN can better capture the complex relationships in the data, among which, CNN’s *A*_cc_, *T*_TP_, and *F*_1_ indicators have reached 97%, which suggests that CNN is able to better capture the data in the transient process spatial features, and compared with MLP and LSTM, CNN has better expressive learning ability.

#### 5.1.3. Performance Comparison of Contrastive Learning

**Figure 7:**
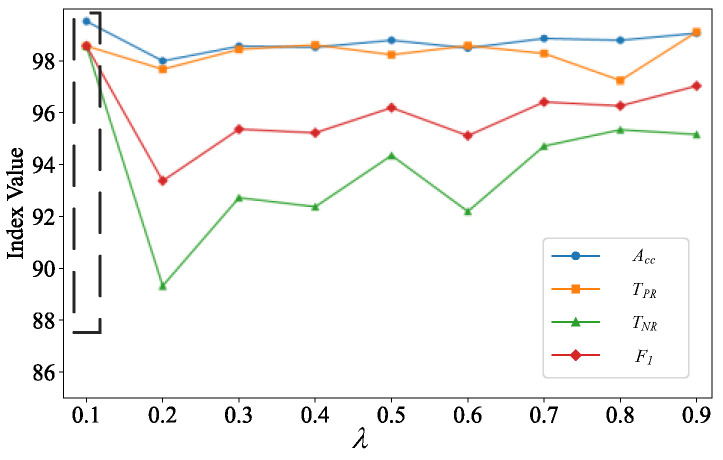
Relationship curves between *λ* and model assessment indices.

To validate the effectiveness of the proposed contrastive learning algorithm, this paper establishes a scenario where the proportion of stable to unstable samples is set to 10:1. This simulates the actual situation of sparse unstable samples, and compares the changes of *A*_cc_, *T*_TP_, *T*_TN_, and *F*_1_ indexes with different *λ* by setting the *λ* balanced cross-entropy loss and comparative loss ratio with the variation range of 0~0.9 and the step size of 0.1. The results of evaluating the role of contrastive learning in assisting training are shown in Figure 7.

As can be seen from Figure 7, when *λ* is 0.1, *A*_cc_, *T*_TP_, *T*_TN_, and *F*_1_ indexes are all above 98, with the gradual increase in *λ*, the evaluation indexes show a certain decline and fluctuation, which indicates that when the contrast loss is too high, it will ignore the features between categories to a certain extent, which affects the model performance. When *λ* is greater than 0.6, the indicators show a certain upward trend, and the *T*_TP_ indicator when *λ* is 0.9 is higher than the *T*_TP_ indicator when *λ* is 0.1, and the *T*_TN_ indicator is lower than the *T*_TN_, indicator when *λ* is 0.1, which indicates that the model’s prediction accuracy is higher for the stable samples when *λ* is 0.9. Considering that more attention is paid to the leakage judgment of the unstable samples in the power system, the *T*_TN_, indicator of the leakage rate is a more important indicator, and for comprehensive consideration, this paper chooses 0.1 as the loss weighting coefficient.

**Figure 8:**
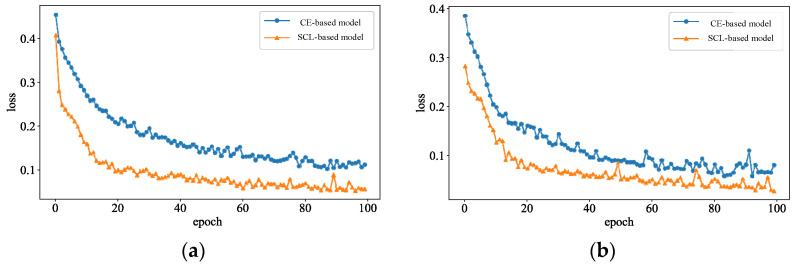
The learning curves of DSA model. (**a**) Training set; (**b**) Test set.

Figure 8 shows the loss function curve of the model incorporating contrast-assisted learning, where Figure 8a shows the test curve on the training set and Figure 8b shows the loss curve on the test set, where the blue curve represents the loss curve of the CNN model using only the cross-entropy function, and the yellow curve represents the loss curve of the CNN model after adding contrast loss. From the figure, the convergence speed of the model is gradually accelerated after adding the contrast-assisted learning, and at 20 cycles, the loss value of the blue curve reaches 0.2, and the loss value of the yellow curve is only 0.1, and the training process of the model is much higher than that of the yellow curve, which says that the contrastive learning can accelerate the convergence of the model, and to some extent, improve the model’s generalization and expressive ability.

#### 5.1.4. Performance Comparison of Offline Training Algorithms

In order to verify the effectiveness of comparison-assisted learning, this paper sets up a variety of sample ratios to simulate the sample imbalance of the actual power grid. The traditional evaluation indexes are directly related to the number of samples in each category, and the indexes are biased towards a larger number of stable samples. In order to cope with this imbalance, which leads to an incomplete evaluation of the indexes, this paper adopts a comprehensive weight-averaging method to calculate the indexes, and the weight-averaging method assigns the same weight to each category to ensure that the evaluation results can respond to the performance of each category in a balanced way. Table 3 shows the testing effect of the algorithm on different ratios.

As can be seen from Table 5, with the gradual increase in the proportion of a stable number of samples, although the *A*_cc_ of the model are kept above 99, the *T*_TN_ indicator in the CNN model, which indicates the accuracy rate of the unstable samples, shows a sustained decline from 97.43 to 80.95, and the *F*_1_, of the comprehensive assessment indicator decreases from 98.44 to 88.03, at which time the model is unable to adequately learn the distributional characteristics of the unstable samples, which is Due to the cross-entropy loss to focus on maximizing the decision boundary between categories, lack of learning the distribution characteristics of intraclass samples. The *T*_TP_ and *F*_1_, metrics of the model incorporating the contrast-assisted learning model under multiple sample imbalance scenarios show substantial improvements. The GNN-based augmented model has lower metrics than the proposed model for multiple sample imbalance ratios. In the scenario with a 40:1 ratio of stable to unstable samples, the fused contrast-assisted learning model reaches over 99.8 for both *A*_cc_ and *T*_TP_, 96.03 for *T*_TN_, and 97.89 for *F*_1_, which indicates that the supervised contrast loss effectively enhances the model’s generalization ability, the contrast loss enhances the model’s perception of the samples within a class by reinforcing the relative distances between the samples. By simultaneously optimizing contrast loss and supervised loss, it helps the model learn more robust feature representations, making it suitable for transient stability assessment of power systems with a sample imbalance.

**Figure 9:**
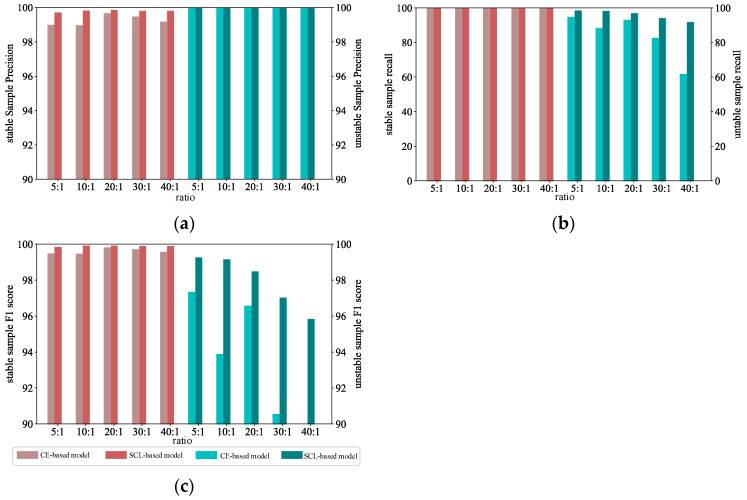
Evaluation of performance of the model on samples of different categories under different sample ratios. (**a**) *T*_TP_; (**b**) *T*_TN_; (**c**) *F*_1_.

To further analyze and compare the boosting effect of the auxiliary module on the unbalanced scenarios, *T*_TP_, *T*_TN_, and *F*_1_ metrics at different ratios are plotted for the stable and unstable samples, respectively. As shown in Figure 9.

The graph shows the indicators for the stable sample in red and the indicators for the unstable sample in green. The stabilized sample performs well in a variety of cases where the sample ratio is not balanced. Indicating each index of the unstable sample, the *T*_TN_ index of the unstable sample decreases to 61.9 when the sample ratio is 40:1, while the index of the model after contrast-assisted for the unstable sample reaches 92.06, which is an enhancement of 48.72%, which proves the enhancement of the contrast-assisted enhancement algorithm for the unstable sample identification.

**Figure 10:**
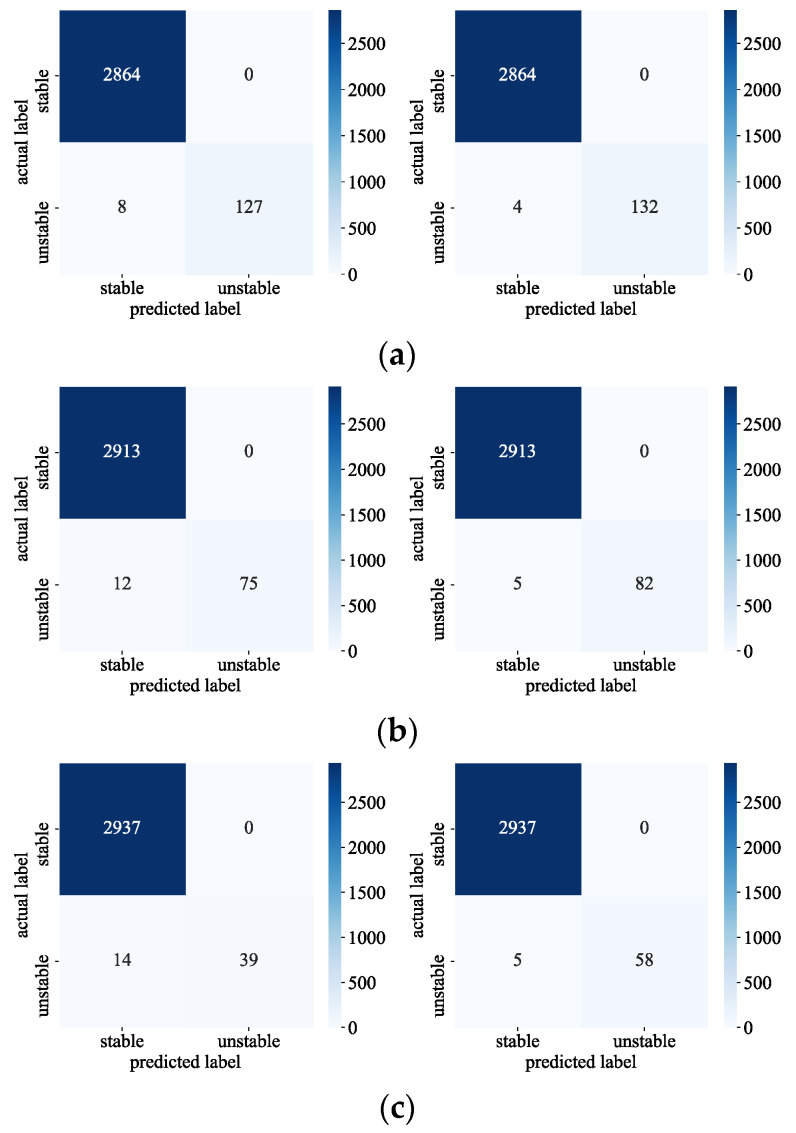
Evaluation of performance of models before and after data augmentation under different proportions. (**a**) Sample imbalance ratio is 20:1; (**b**) Sample imbalance ratio is 30:1; (**c**) Sample imbalance ratio is 40:1.

Figure 10 plots the model evaluation results of the contrast-assisted module before and after enhancement at different sample ratios. When the ratio of stable destabilizing samples is 20:1, compared with the model before enhancement, the missed samples of the enhanced model are reduced from 8 to 4, and the recognition accuracy of destabilizing samples is improved by 50%; when the ratio of stable destabilizing samples is 20:1, compared with the model before enhancement, the missed samples of the enhanced model are reduced from 12 to 5, and the recognition accuracy of destabilizing samples is improved by 58% when the ratio of stable destabilizing samples is 30:1, demonstrating that the proposed method proves more effective in recognizing destabilizing samples improved by 58%, demonstrating that the proposed method significantly enhances the model’s recognition rate of unstable samples.

**Figure 11:**
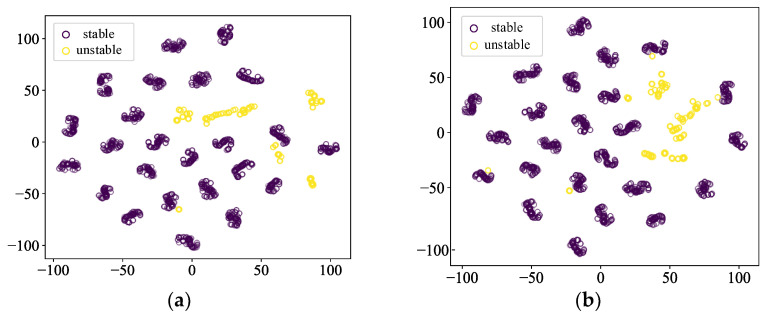
Comparison of feature distributions when the sample imbalance ratio is 5. (**a**) CE-based model (**b**) SCL-based model.

**Figure 12:**
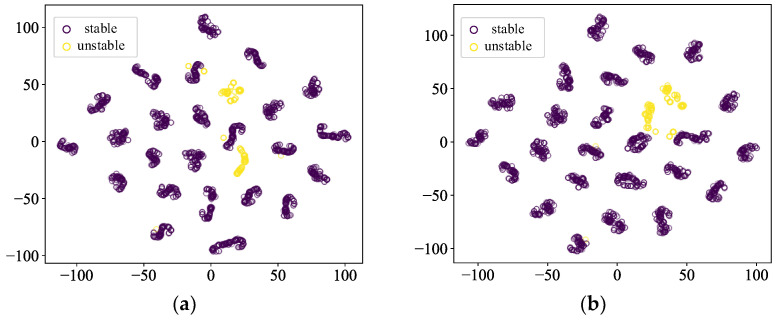
Comparison of feature distributions when the sample imbalance ratio is 10. (**a**) CE-based model (**b**) SCL-based model.

The t-SNE method is used in making spatial distribution maps of the sample features in this paper, and the purple circles indicate the stable samples, and the yellow circles indicate the unstable samples. Figure 11 and Figure 12 show the scenarios where the ratio of stable samples to unstable samples is 5:1 and 10:1, respectively. The yellow circles indicating unstable samples in the model with the addition of the contrastive learning show a tendency to be more clustered, which suggests that contrastive learning effectively captures the similarities and differences between the data, and improves the model’s expressive ability.

### 5.2. Comparison of the Effectiveness of Active Transfer Methods

In order to verify the practicality of the algorithm, this paper adds two target scenarios considering fluctuations in new energy output and maintenance of line equipment to the initial setup in Section 5.1 to simulate the changing operating conditions during the online application. The source system is set as a high permeability system with 60–80% new energy output; the target system 1 is a low permeability system with 20–40% new energy output, and the target system 2 is a high permeability system with 110%, 115%, and 120% heavy loads, and 60–80% new energy output; the thermal power generator outputs are adjusted accordingly to ensure convergence of the tidal current calculations in the above systems. The fault conditions are set up in the same way as the initial fault set of the IEEE39 node system. A new target domain dataset is produced via the time domain simulation method for verifying the performance of the model based on the source domain training on the target domain dataset. Results are presented in Table 6.

The source domain model has a high evaluation accuracy on the dataset of the source domain, while evaluation inaccuracy rises significantly when applied directly to the target system with structural changes, suggesting that the model needs to be migrated at this point in order to increase the evaluation performance.

To validate the generalization of the SCL-based model proposed in this paper for new scenarios, it is compared with the CE-based model, both of which are the CNN models proposed above. Table 7 shows the comparison of the generalization ability of the models on the target system. From the table, it can be seen that the SCL-based model outperforms the CE-based model in all metrics and has better generalization performance. This indicates that contrastive learning contributes to the robustness of the model to features, which can improve the robustness at different data distributions and is more suitable to be used as a benchmark model for transfer.

**Figure 13:**
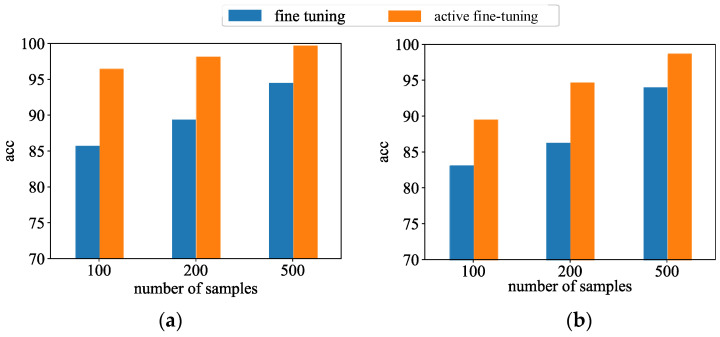
Comparison of different active transfer learning strategies for target system. (**a**) Target domain 1; (**b**) Target domain 2.

This paper tests the performance of the model over different target domain datasets to verify the effectiveness of the knot active transfer strategy. Figure 13 illustrates the performance compared to the prediction performance between the active fine-tuning learning method and the random sample fine-tuning method under different sample numbers. It is obvious from the figure that the prediction performance of the proposed active fine-tuning learning method is significantly better than that of the random sample fine-tuning method, especially if the sample size is smaller. When 100 samples were selected for updating, the active fine-tuning learning method was found to be substantially more accurate than the random sample fine-tuning learning method, with a specific improvement of 12.56%. It is further shown that active learning is able to selectively pick samples with important information, which enables the model to achieve excellent performance despite limited data conditions.

## 6. Conclusions and Future Work

Aiming at the problems of sample data imbalance and poor generalization in machine learning-based transient stability assessment, a real-time adaptive assessment method for transient stability with intelligent enhancement of models is proposed. It is validated on an IEEE39 node system, and the conclusions obtained are as follows:

(1) The training algorithm based on supervised contrastive learning helps the model to learn more robust feature representations, which can effectively improve the accuracy of the model in recognizing unbalanced samples and perform well under multiple unbalanced ratios. The t-SNE-based visualization indicates that the proposed method effectively captures the similarities and differences between the data, enhancing the representational capacity of the feature space.

(2) Active learning based on uncertainty sampling can quickly select a small number of the most informative samples. The joint training of active learning and transfer learning can significantly improve the generalization ability of the DSA model in new scenarios, while effectively reducing the cost of model updates.

The proposed method effectively improves the model’s ability to recognize unstable samples in the presence of sample imbalance. However, the current data-driven method still cannot completely eliminate the problem of misjudging unstable samples as stable samples, which limits its applicability in practical application scenarios. In addition, the common voltage instability problem in actual grid operation needs more attention. In future work, further in-depth exploration will be conducted in subsequent studies to enhance the applicability of the data-driven method.

## Figures and Tables

**Table 1:** Feature subset.

Feature Subset	Feature Description
Feature subset I	active power and reactive power of transmission line
Feature subset II	active power and reactive power of the transmission line, as well as bus voltage magnitude and phase angle
Feature subset III	active and reactive power of transmission lines, active and reactive power of generators, and active and reactive power of loads
Feature subset IV	active and reactive power of transmission lines, bus voltage magnitude, phase angle, active and reactive power of generators, and active and reactive power of loads

**Table 2:** Confusion matrix.

	Stable (Predicted)	Unstable (Predicted)
stable (actual)	TP	FP
unstable (actual)	FN	TN

**Table 3:** Feature subset performance.

	*A*_cc_	*T*_TP_	*T*_TN_	*F*_1_
Feature subset 1	94.33	90.91	95.10	92.71
Feature subset 2	97.90	98.43	95.88	97.08
Feature subset 3	97.80	97.31	96.69	97.00
Feature subset 4	96.87	96.83	94.60	95.65

**Table 4:** Model evaluation performance.

Model	*A*_cc_	*T*_TP_	*T*_TN_	*F*_1_
MLP	97.00	97.17	94.64	95.82
LSTM	96.50	97.43	93.11	95.04
CNN	97.90	98.43	95.88	97.08
RF	95.47	95.46	95.48	95.47

**Table 5:** Evaluation performance of models under different sample proportions.

	CNN				CNN Based on CL				CNN Enhanced by GNN			
Ratio	*A*_cc_	*T*_TP_	*T*_TN_	*F*_1_	*A*_cc_	*T*_TP_	*T*_TN_	*F*_1_	*A*_cc_	*T*_TP_	*T*_TN_	*F*_1_
5:1	99.17	99.51	97.43	98.44	99.77	99.86	99.28	99.57	98.94	98.93	98.95	98.94
10:1	99.07	99.50	94.26	96.70	99.87	99.93	99.18	99.55	98.75	98.77	98.77	98.75
20:1	99.70	99.84	96.69	98.21	99.87	99.93	98.53	99.22	99.09	99.09	99.09	99.09
30:1	99.50	99.74	91.38	95.15	99.83	99.91	97.13	98.48	98.90	98.93	98.89	98.90
40:1	99.20	99.59	80.95	88.03	99.83	99.92	96.03	97.89	99.28	99.29	99.28	99.28

**Table 6:** Testing results of original model in target systems.

	Training Set		Test Set	
	Number of Samples	*A*_cc_	Number of Samples	*A*_cc_
Source domain	7000	97.49	3000	97.47
Target domain1	7000	83.38	3000	83.22
Target domain2	7000	81.53	3000	79.35

**Table 7:** Testing results of the original model in target systems.

	Model	*A*_cc_	*T*_TP_	*T*_TN_	*F*_1_
Target domain1	CE-based model	73.91	67.62	72.01	68.56
	SCL-based model	83.22	77.05	75.94	76.46
Target domain2	CE-based model	74.07	73.97	75.20	73.72
	SCL-based model	79.35	78.86	77.81	78.21

## Data Availability

No new data were created or analyzed in this study. Data sharing is not applicable to this article.

## References

[1] Guo Y., Hill D.J., Wang Y. (2001). Global transient stability and voltage regulation for power systems. IEEE Trans. Power Syst..

[2] Amjady N., Majedi S.F. (2007). Transient stability prediction by a hybrid intelligent system. IEEE Trans. Power Syst..

[3] Chu S., Majumdar A. (2012). Opportunities and challenges for a sustainable energy future. Nature.

[4] Ci L., Sun Y., Chen X. (2012). Preliminary analysis of large scale blackout in Western Europe power grid on November 4 and measures to prevent large scale blackout in China. IEEE Trans. Power Syst. Technol..

[5] Cremer J.L., Konstantelos I., Strbac G. (2019). From optimization-based machine learning to interpretable security rules for operation. IEEE Trans. Power Syst..

[6] Kumbhar A., Dhawale P.G., Kumbhar S., Patil U., Magdum P. (2021). A comprehensive review: Machine learning and its application in integrated power system. Energy.

[7] Sarajcev P., Kunac A., Petrovic G., Despalatovic M. (2022). Artificial intelligence techniques for power system transient stability assessment. Energies.

[8] Zhu Q., Chen J., Zhu L., Shi D., Bai X., Duan X., Liu Y. (2018). A deep end-to-end model for transient stability assessment with PMU data. IEEE Access.

[9] Yi J., Lin W., Hu J., Dai J., Zhou X., Tang Y. (2020). An integrated model-driven and data-driven method for on-line prediction of transient stability of power system with wind power generation. IEEE Access.

[10] Tsotsopoulou E., Karagiannis X., Papadopoulos P., Dyśko A., Yazdani-Asrami M., Booth C., Tzelepis D. (2022). Time-domain protection of superconducting cables based on artificial intelligence classifiers. IEEE Access.

[11] Bo W., Fang B., Wang Y. (2016). Power System Transient Stability Assessment Based on Big Data and the Core Vector Machine. IEEE Trans. Smart Grid.

[12] Fan Y., Li X., Zhang P. (2017). Integrated approach for online dynamic security assessment with credibility and visualization based on exploring connotative associations in massive data. IEEE Access.

[13] Liu S., Liu L., Fan Y. (2020). An Integrated Scheme for Online Dynamic Security Assessment Based on Partial Mutual Information and Iterated Random Forest. IEEE Trans. Smart Grid.

[14] Zhang T., Sun M., Cremer J.L., Zhang N., Strbac G., Kang C. (2021). A confidence-aware machine learning framework for dynamic security assessment. IEEE Trans. Power Syst..

[15] Ren C., Yuan H., Li Q., Zhang R., Xu Y. (2021). Pre-Fault Dynamic Security Assessment of Power Systems Handling Multiple Fault Types via Multi-Label Learning. IEEE Trans. Power Syst..

[16] Ngo T.A., Nguyen T., Thang T.C. (2023). A survey of recent advances in quantum generative adversarial networks. Electronics.

[17] Kumar S., Kansal S., Alkinani M.H., Elaraby A., Garg S., Natarajan S., Sharma V. (2022). Segmentation of spectral plant images using generative adversary network techniques. Electronics.

[18] Asre S., Anwar A. (2022). Synthetic energy data generation using time variant generative adversarial network. Electronics.

[19] Li Y., Zhang M., Chen C. (2022). A deep-learning intelligent system incorporating data augmentation for short-term voltage stability assessment of power systems. Appl. Energy.

[20] Zhan X., Han S., Rong N., Cao Y. (2023). A hybrid transfer learning method for transient stability prediction considering sample imbalance. Appl. Energy.

[21] Liu B., Yu H., Du J., Wu Y., Li Y., Zhu Z., Wang Z. (2022). Specific emitter identification based on self-supervised contrast learning. Electronics.

[22] Li X., Zhao Z., Zhang Y., Zheng S., Dai S. (2023). Spectrum sensing algorithm based on self-supervised contrast learning. Electronics.

[23] Zhu H., Chen Y., Hu G., Yu S. (2022). Contrastive learning via local activity. Electronics.

[24] Fornás J.G., Jaraba E.H., Estopiñan A.L., Saldana J. (2022). Detection and classification of fault types in distribution lines by applying contrastive learning to GAN encoded time-series of pulse reflectometry signals. IEEE Access.

[25] Liu M., Liu C., Fu X., Wang J., Li J., Qi Q., Liao J. (2023). Deep Clustering by Graph Attention Contrastive Learning. Electronics.

[26] Ranaweera M., Mahmoud Q.H. (2021). Virtual to real-world transfer learning: A systematic review. Electronics.

[27] Xie K., Wang C., Wang P. (2021). A domain-independent ontology learning method based on transfer learning. Electronics.

[28] Weiss K., Khoshgoftaar T.M., Wang D. (2016). A survey of transfer learning. J. Big Data.

[29] Ren C., Xu Y., Dai B., Zhang R. (2021). An integrated transfer learning method for power system dynamic security assessment of unlearned faults with missing data. IEEE Trans. Power Syst..

[30] Xie J., Sun W. (2021). A transfer and deep learning-based method for online frequency stability assessment and control. IEEE Access.

[31] Gunel B., Du J., Conneau A., Stoyanov V. Supervised Contrastive Learning for Pre-trained Language Model Fine-tuning. Proceedings of the International Conference on Learning Representations.

[32] Khosla P., Teterwak P., Wang C., Sarna A., Tian Y., Isola P., Maschinot A., Liu C., Krishnan D. (2020). Supervised contrastive learning. Adv. Neural Inf. Process. Syst..

[33] Yosinski J., Clune J., Bengio Y., Lipson H. (2014). How transferable are features in deep neural networks?. Adv. Neural Inf. Process. Syst..

